# Analysis of tiling array expression studies with flexible designs in Bioconductor (waveTiling)

**DOI:** 10.1186/1471-2105-13-234

**Published:** 2012-09-14

**Authors:** Kristof De Beuf, Peter Pipelers, Megan Andriankaja, Olivier Thas, Dirk Inzé, Ciprian Crainiceanu, Lieven Clement

**Affiliations:** 1 Department of Mathematical Modelling, Statistics and Bioinformatics, Ghent University, Coupure Links 653, B9000 Ghent, Belgium; 2 Department of Plant Systems Biology, Flanders Institute for Biotechnology, Ghent, Belgium; 3 Department of Plant Biotechnology and Bioinformatics, Ghent University, Ghent, Belgium; 4 Department of Biostatistics, Johns Hopkins Bloomberg School of Public Health, 615 N. Wolfe St., Baltimore, MD, USA; 5 Interuniversity Institute for Biostatistics and Statistical Bioinformatics, Katholieke Universiteit Leuven and Universiteit Hasselt, Kapucijnenvoer 35, Blok D, bus 7001B3000 Leuven, Belgium

## Abstract

**Background:**

Existing statistical methods for tiling array transcriptome data either focus on transcript discovery in one biological or experimental condition or on the detection of differential expression between two conditions. Increasingly often, however, biologists are interested in time-course studies, studies with more than two conditions or even multiple-factor studies. As these studies are currently analyzed with the traditional microarray analysis techniques, they do not exploit the genome-wide nature of tiling array data to its full potential.

**Results:**

We present an R Bioconductor package, waveTiling, which implements a wavelet-based model for analyzing transcriptome data and extends it towards more complex experimental designs. With waveTiling the user is able to discover (1) group-wise expressed regions, (2) differentially expressed regions between any two groups in single-factor studies and in (3) multifactorial designs. Moreover, for time-course experiments it is also possible to detect (4) linear time effects and (5) a circadian rhythm of transcripts. By considering the expression values of the individual tiling probes as a function of genomic position, effect regions can be detected regardless of existing annotation. Three case studies with different experimental set-ups illustrate the use and the flexibility of the model-based transcriptome analysis.

**Conclusions:**

The waveTiling package provides the user with a convenient tool for the analysis of tiling array trancriptome data for a multitude of experimental set-ups. Regardless of the study design, the probe-wise analysis allows for the detection of transcriptional effects in both exonic, intronic and intergenic regions, without prior consultation of existing annotation.

## Background

In the last few years tiling microarrays have become a well-established tool for whole-genome transcriptome analysis. They have shown to be very useful for exploring and unraveling the complex genome-wide trancriptional landscape of higher organisms, in which not only protein coding genes, but also non-coding RNAs play an important role [1-4]. The methods that have been developed for transcriptome analysis with tiling arrays either focus on segmentation and transcript discovery within a single biological condition [5-8], or on the detection of differential expression between two distinct conditions [9,10]. Recently, the focus in tiling array studies has shifted towards more complex experimental designs, such as studies with more than two conditions [11] and studies with several experimental factors [12]. Furthermore, it is recognized that expression is a dynamic rather than a static phenomenon. Hence, more and more time-course experiments are designed to provide insights into the whole-genome transcript regulation of species during different developmental stages or external periodic changes in the environment [13,14].

Currently, most tiling array transcriptome analysis pipelines start with summarization of the probe-level data. This can be done by constructing probesets from the groups of probes that map to known annotated genes, (e.g. [11,15]). Hereby unannotated regions are disregarded. In [12,13,16] a sliding window-based approach is adopted, combined with a thresholding rule for selecting transcriptional units, whereas in [14] segments with piece-wise constant intensity levels are constructed first [17]. After the summarization a statistical test or a more heuristic analysis technique is conducted on the summarized expression values of the transcriptional units. In current time-course and single-factor studies this is merely done by directly applying traditional microarray analysis methods, such as a pairwise moderated t-test (Limma) [18] conducted in [11] or a permutated t-test (SAM) [19] conducted in [16]. Other studies adopt ad-hoc approaches to filter the genes or transcriptional units of interest. Transcriptional units in a time-course experiment, for example, can be filtered based on thresholding the amplitude of the signal [20]. In an alternative approach the correlations between temporal expression patterns are explored and a clustering is performed of genomic regions based on expression profiles in different gene classes showing expression at different time-points [21]. The tests reported in [13] and [14] on the other hand are less ad hoc, but very specific for the periodic time-course design apparent in these studies [22-24]. The aforementioned methods either lack flexibility by only focusing on one specific experimental design, or they first summarize probes to probesets based on existing annotation, hence not exploiting the genome-wide nature of the data to the full extent.

Here, we present waveTiling, a R Bioconductor package for transcriptome analysis of tiling arrays with flexible designs. The package is based on and provides an extension to a recently introduced wavelet-based functional model for transcriptome analysis [25]. While the methodology in [25] was initially developed to conduct the simultaneous tasks of transcript discovery and detection of differential expression, their framework can be easily extended by adapting the model design matrix. After modeling the specific effect function of interest, probe-wise inference can be conducted for detecting affected regions. The probe-wise analysis allows for the detection of transcriptional units in both exonic, intronic and intergenic regions, without prior consultation of existing annotation. Currently, waveTiling provides a standard analysis flow for transcriptome analysis on single-factor experiments with two or more biological conditions, the detection of linear and quadratic effects and circadian rhythms in time-course experiments, and the analysis of two-factor experiments, while more experienced users can also specify customized designs. Furthermore, it generates along-genome plots and contains functions to easily extract the detected genes and unannotated regions. The *Implementation* section gives an overview of the main functionalities of the waveTiling package and describes the model for the different designs, as well as the associated inference procedures. In *Results and Discussion* we illustrate the use of the package and the model on three different case studies with very distinct experimental designs.

## Implementation

The waveTiling package is an add-on package to the Bioconductor project [26] written in the programming language and statistical environment R [27]. It provides all the tools necessary to conduct a full analysis of tiling microarray experiments for flexible designs based on the recently introduced wavelet-based functional model for trancriptome analysis [25]. The package uses the standard Bioconductor S4-class data structures making it fully compatible with existing packages. The data is imported with the aid of the oligo-package [28] and the resulting object inherits from *TilingFeatureSet*, which is specifically designed for representing tiling array data and in turn extends *ExpressionSet*. Existing instance methods from oligo and other Bioconductor packages supporting this structure are therefore applicable as well. Before starting the analysis the probes can be remapped to the existing annotation. Moreover, probes that contain duplicated sequences for perfect match and mismatch probes or for probes on different strands can be filtered because they are deemed unreliable due to cross-hybridization effects. The main transcriptome analysis consists of two consecutive steps: (1) fitting the wavelet-based functional model to the data, and (2) model-based inference to identify transcriptionally affected regions. The fitted model is stored in a *WfmFit*-class object. Depending on the design of the study a *WfmFitFactor* (factorial design), *WfmFitTime* (time-course design), *WfmFitCircadian* (circadian rhythm design) or *WfmFitCustom* (custom design) subclass is used. Part of the code for fitting the model is implemented in C to speed up computation. In the second step, different inference procedures can be conducted depending on the research question. The inference procedure that can be conducted depends on the *WfmFit*-subclass. The results are stored as a *WfmInf *-class object. There are 3 main subclasses: *WfmInfCompare* which contains the results of a pairwise comparison between two groups or time points; *WfmInfMeans* with the results of transcript discovery for each individual group or time point; and *WfmInfEffects* which contains results with linear or quadratic time effects for time-course designs and circadian rhythm effects for circadian designs. All transcriptionally affected regions can be extracted from the *WfmInf *-class objects and are stored as *IRanges*-class objects [29]. The model fitting and inference steps are described in more detail in the *Statistical Methods* part.

The results can be visually explored by means of a general plot function. The implementation is based on the GenomeGraphs package [30]. For any genomic region the fitted expression values and transcriptionally affected regions can be plotted along the genomic coordinate. Furthermore, two functions are available for further post-processing of the results. Provided a suitable annotation file is given, the transcriptionally affected regions are mapped against the existing annotation. The first function outputs the genes that are transcriptionally affected, while the second function provides a list of the detected unannotated regions. The output of both functions is a list of *GRanges*-class objects [31].

## Statistical Methods

We start by presenting an overview of the basic model introduced by [25]. Subsequently, we show how we accomodate for several sampling schemes in time-course experiments or other experiments with more flexible designs.

### Basic wavelet-based model for transcriptome analysis

We consider the functional model designed for the detection of (differentially) expressed regions in experiments with two biological conditions. It is given by [25]

(1) $$Y_{i}(t)=\beta_{1}(t)+X_{1,i}\beta_{2}(t)+E_{i}(t),$$

with *i*=1,*..N*, _*Yi*_(*t*) the measured log_2_-transformed expression values for the probe with position *t* (*t*=1,*..T*) on array *i* (*i*=1,*..N*). *T* is the number of probes that are more or less equally spaced along the genomic position of the chromosome, and *N*=_*N*1_ + _*N*2_ is the number of tiling arrays in the experiment, with _*N*1_ the number for biological condition 1, say _*C*1_, and _*N*2_ the number for biological condition 2, say _*C*2_. Further, _*X*1,*i*_ is a dummy variable which is 1 for _*C*1_and −1 for _*C*2_, and _*Ei*_(*t*) is a zero mean error term. It is assumed that _*Ei*_(1),*..*_*Ei*_(*T*) are jointly *MVN*(***0***_***Σ****ε*_). Here, *MVN*(***μΣ***) denotes the density function of a multivariate normal distribution with mean ***μ*** and variance-covariance matrix ***Σ***.

The model can also be written as

(2) Y=XB+E.

In this model, ***Y***is an *N*×*T*matrix of measured log_2_-transformed expression values, containing the elements _*Yi*_(*t*) for the probe with genomic position *t* (*t*=1,*.*,*T*) on array *i* (*i*=1,*..*,*N*). Further, ***E*** is an *N*×*T* error matrix containing the errors terms _*Ei*_(*t*) for probe position *t* on array *i*. The *N*×2 design matrix ***X***is constructed as

$$X=\left[\begin{array}{c} 1\;1\\ 1\;\;\;\;-1 \end{array}\right],$$

where the upper row represents the dummy coding for the _*N*1_ arrays in _*C*1_ and the lower row the dummy coding for the _*N*2_ arrays in _*C*2_. The 2×*T* effect function matrix ***B*** contains the probe-wise effect functions _*β*1_(*t*) and _*β*2_(*t*) on the respective rows. Column 1 of ***X***will be used to find regions with a mean expression level above some threshold, whereas the coding in column 2 allows for assessing differential expression between the two conditions. Note that the coding in ***X***implies that two effect functions are estimated orthogonally for a balanced study design. This can be seen from

$$X^{T}X=\left[\begin{array}{c} N/2\;0\\ \;\;\;0\;N/2 \end{array}\right],$$

with $N_{1}=N_{2}=\frac{N}{2}$.

Before estimating the effect functions, the expression data are projected onto the wavelet space by using the discrete wavelet transform (DWT). This linear projection can be written as the matrix multiplication ***D***=***Y***^***W****T*^, where ***W*** is an orthogonal DWT matrix. This allows us to rewrite model (2) in the wavelet space as

(3) $$D=XB^{*}+E^{*},$$

where the rows of the *N*×*T*matrix ***D***contain the wavelet coefficients for each array, double-indexed by location *k*=1,*..*_*Kj*_ and scale *j*=0,*..J*. The 2×*T* and *N*×*T* matrices ^***B***∗^ and ^***E***∗^ contain the wavelet coefficients for the effect functions and the error terms, respectively. By putting a normal prior on the effect functions in the wavelet space, this model can also be written as

(4) $$\left\{\begin{array}{ccc} D(j,k)|\beta^{*}(j,k) & ∼ & \;\;\;\mathrm{MVN}\left\{{\mathrm{X\beta}}^{*}(j,k),I\sigma_{\varepsilon }^{2}(j,k)\right\}\\ \beta_{m}^{*}(j,k)|\tau_{m}(j,k) & ∼ & \;\;\;N\left\{0,\tau_{m}(j,k)\sigma_{\varepsilon }^{2}(j,k)\right\} \end{array}\right),$$

where $\beta_{m}^{*}(j,k)$ is the element of ^***B***∗^ corresponding to scale *j* and location *k* and *m*=1,2. In (4) *N*(*μ*^*σ*2^) denotes the density function of a normal distribution with mean *μ*and variance ^*σ*2^. The smoothing parameters _*τm*_(*jk*) and the error variances $\sigma_{\varepsilon }^{2}(j,k)$ are estimated by marginal maximum likelihood using a Gauss-Seidel algorithm. The estimated ${\hat{\tau }}_{m}(j,k)$ induce a regularization of the wavelet coefficients of the effect functions. When backtransforming the modified coefficients to the original data space, this leads to a denoised expression signal whereby the main features are retained. The method has proven to be very fast which is essential when analyzing large datasets. For more details, see [25].

### Wavelet-based models for transcriptome analysis in more flexible designs

To extend the modeling framework reviewed in the previous section and to make it suitable for the analysis of tiling array data with more flexible designs, the design matrix ***X*** needs to be adapted in an appropriate way. Firstly, the adaptation must enable the model to answer the specific research questions provoked by the experimental design. Secondly, it must allow us to use the same fast algorithms introduced in [25]. This second argument comes down to the preservation of the orthogonality of ***X***. In the first part of this section we focus on general time-course designs and single-factor designs for more than 2 groups. The second part aims at specific time-course designs for assessing circadian rhythms in the transcriptome. The section concludes with looking specifically at non-orthogonal designs, typically encountered in multi-factor studies.

#### General time-course designs

In tiling array time-course experiments one is often interested in the detection of differentially expressed regions between any two different time points. An additional concern might be to detect significant effects of transcriptional activity in time, e.g. linearly increasing or decreasing transcriptional expression of certain regions. These two possible research aims can be dealt with by considering a functional relationship of the designed time points described by orthogonal polynomials. This approach has also been used in quantitative trait associated expression studies based on traditional microarrays [32]. In that paper the functional relationship with phenotype is considered instead of with time.

Consider a time-course experiment with whole-genome expression levels measured at *q* time points. Let *N* be the total number of arrays used in the experiment. The number of arrays used for each time point is represented by _*N*1_,…,_*Nq*_, with _*N*1_ + … + _*Nq*_=*N*. In this exposition we only consider balanced designs, i.e. _*N*1_=…=_*Nq*_, with equidistant time points. However, it is rather straightforward to obtain orthogonal polynomials when dealing with non-balanced and non-equidistance designs. A simple procedure is discussed in [33]. The design matrix ***X*** in model (2) now has dimensions *N*×*q*and can be written as

(5) $$X=\left[\begin{array}{ccccc} 1 & \psi_{1}(X_{1}) & \;\psi_{2}(X_{1}) & \ldots & \;\psi_{q-1}(X_{1})\\ 1 & \psi_{1}(X_{2}) & \;\psi_{2}(X_{2}) & \ldots & \;\psi_{q-1}(X_{2})\\ \;\vdots & \;\;\;\;\vdots \;\;\; & \;\;\;\vdots \;\;\;\; & \;\vdots \;\;\; & \;\;\;\;\;\;\vdots \\ 1 & \psi_{1}(X_{q}) & \;\psi_{2}(X_{q}) & \ldots & \;\psi_{q-1}(X_{q}) \end{array}\right],$$

where $X_{1},\ldots ,X_{q}$ are the _*N*1_,…,_*Nq*_-valued vectors that correspond with the *q* respective designed time points in the experiment. In (5) each function _*ψj*_(***x***) is a polynomial of degree *j*, with *j*=0,…,*q*−1, and is orthogonal to _*ψk*_(***x***) (*k*=0,…,*q*−1) if *j*≠*k*. Note that each ***1*** in the first column of ***X*** can also be seen as _*ψ*0_(_***X****i*_) ( i=1,…,q). The orthogonality of ***X***is clear from

(6) $$\;X^{T}X\;=\;\left[\;\;\;\;\begin{array}{llllll} N & 0 & 0 & \;\;0 & \ldots & 0\\ 0 & \;\;\overset{N}{\underset{i=1}{\sum }}\overset{2}{\underset{1}{\psi }}(X_{i}) & 0 & \;\;0 & \ldots & 0\\ 0 & 0 & \;\;\overset{N}{\underset{i=1}{\sum }}\overset{2}{\underset{2}{\psi }}(X_{i}) & \;\;0 & \ldots & 0\\ \vdots & \vdots & \vdots & \;\;\vdots & \vdots & \vdots \\ 0 & 0 & 0 & \;\;\ldots & \;\overset{N}{\underset{i=1}{\sum }}\overset{2}{\underset{q-2}{\psi }}(X_{i}) & 0\\ 0 & 0 & 0 & \;\;\ldots & 0 & \;\;\overset{N}{\underset{i=1}{\sum }}\overset{2}{\underset{q-1}{\psi }}(X_{i}) \end{array}\;\;\;\;\right]\;.$$

With this design matrix a *q*×*N*matrix ***B*** with the *q* effect functions is associated. The first row of ***B***corresponds with an overall mean expression level over all time points, while row 2 until *q* are associated with a linear, quadratic, cubic,…,(*q*−1)-th order polynomial effect respectively between the different time points. The fitted expression levels for each time point are obtained by a linear combination of all effect functions in accordance with model (2). This allows for a straightforward comparison between any two time points. When combining several effect functions, it may be desirable to induce the same amount of smoothing for each of them. This implies the estimation of one general smoothing parameter *τ*(*jk*), instead of a separate _*τm*_(*jk*) for each effect function (*m*=1,*..q*). To retain the fast algorithms of [25], however, the diagonal elements of ^***X****T*^***X***need to be identical in this case. This can be obtained by normalizing each column vector of ***X*** to give the normalized design matrix ***^*X″*^***. This leads to the property

(7) $${X^{\prime \prime }}^{T}X^{\prime \prime }=\left[\begin{array}{cccccc} 1 & 0\; & 0 & 0\; & \ldots & 0\\ 0 & 1\; & 0 & 0\; & \ldots & 0\\ 0 & 0\; & 1 & 0\; & \ldots & 0\\ \vdots & \vdots \; & \vdots & \vdots \; & \;\;\;- & \vdots \;\\ 0 & 0\; & 0 & \ldots \; & \;\;\;1 & 0\\ 0 & 0\; & 0 & \ldots \; & \;\;\;0 & 1 \end{array}\right]=I_{q},$$

where _***I****q*_ is an *q*×*q* identity matrix. For this orthonormal design matrix ***^*X″*^***it can be shown that the common smoothing parameter can be estimated by

(8) $$\hat{\tau }(j,k)={\left[\frac{D^{T}(j,k)X^{\prime \prime }{X^{\prime \prime }}^{T}D(j,k)}{q\sigma_{\varepsilon }^{2}(j,k)}\right]}_{+}.$$

Although design matrix (5) can also be used for non-ordered single factor studies, one may choose to use a design matrix specifically constructed for unordered factors, e.g. a Helmert contrast design matrix. Helmert contrasts are basically designed to compare the mean expression at a specific time point with the overall mean over all preceding time points. The main reason why we use them here, however, is that they also lead to estimation orthogonalities for the effect functions. This is seen from

(9) $$X^{T}X=\left[\begin{array}{llllll} N & 0 & 0 & 0 & \ldots & 0\\ 0 & \overset{2}{\underset{i=1}{\sum }}N_{i} & 0 & 0 & \ldots & 0\\ 0 & 0 & 2\overset{3}{\underset{i=1}{\sum }}N_{i} & 0 & \ldots & 0\\ \vdots & \vdots & \vdots & \vdots & \vdots & \vdots \\ 0 & 0 & 0 & \ldots & (q-2)\overset{q-1}{\underset{i=1}{\sum }}N_{i} & 0\\ 0 & 0 & 0 & \ldots & 0 & (q-1)\overset{q}{\underset{i=1}{\sum }}N_{i} \end{array}\right].$$

Just like for the polynomials, the design matrix ***X***based on Helmert contrasts still needs to be normalized if the same smoothing for all factor effects is desired.

#### Designs for circadian rhythms

Suppose now that we are interested in the detection of a certain circadian rhythm in the transcriptome of an organism, based on an equally spaced time-course experiment. A natural way to model the circular effect is to construct ***X*** by means of Fourier basis functions, instead of polynomial basis functions. The design matrix is then given by

(10) $$X=\left[\begin{array}{ccc} 1 & \text{sin}(0)\;\;\;\; & \;\text{cos}(0)\\ 1 & \text{sin}(\frac{2\Pi }{q})\; & \;\text{cos}(\frac{2\Pi }{q})\\ 1 & \text{sin}(\frac{4\Pi }{q})\; & \;\text{cos}(\frac{4\Pi }{q})\\ \vdots & \vdots \;\;\;\;\;\; & \;\;\;\;\;\;\;\;\;\;\vdots \\ 1 & \text{sin}(2\Pi -\frac{\Pi }{q}) & \;\text{cos}(2\Pi -\frac{\Pi }{q}) \end{array}\right]$$

Again the separate effect functions can be estimated orthogonally, which is seen from

(11) $$X^{T}X=\left[\begin{array}{ccc} N & 0\;\;\; & 0\\ 0 & q\;\;\; & 0\\ 0 & 0\;\;\; & q \end{array}\right].$$

To estimate a common smoothing parameter for inducing the same amount of smoothing for all effect functions, ***X*** can again be normalized as described previously.

#### Non-orthogonal designs

Design matrices for two- or multiple-factor designs are typically non-orthogonal. Using these in the wavelet-based model would imply that the fast algorithms presented in [25] would have to be adapted. This would lead to undesirably increased computation time during parameter estimation. A solution to this problem is to apply the Gram-Schmidt process to orthogonalize ***X***and subsequently estimate the model parameters based on the orthogonalized design matrix. The Gram-Schmidt orthogonalization comes down to a QR-decomposition [34] of ***X*** into an upper-triangular matrix _***X****tri*_ and an orthogonal matrix _***X****orth*_, which is now used to fit the model. Afterwards, the estimated parameters have to be transformed back to obtain the parameter values for the original ***X***. This is possible by premultiplying them with ${\left(X_{\mathrm{orth}}^{T}X\right)}^{-1}$. Similar to single-factor and time-course designs, the coding of the initial design matrix ***X*** still determines how the parameters can be interpreted, and may thus be constructed according to the specific research interest.

### Statistical inference: detection of transcriptional effect regions

Depending on the study design and the aim of the analysis, either the parameters themselves or a function of the parameters are used to detect transcriptional effect regions. In both instances, the effect of interest can be represented by $F\left\{\beta (t)\right\}$. For general time-course designs one can be interested in detecting genomic regions that show a linear or a quadratic trend in time. In this situation $F\left\{\beta (t)\right\}$ is just the effect function ***β***(*t*) that corresponds with either the linear polynomial term _*ψ*1_(***X***) or the quadratic polynomial term _*ψ*2_(***X***) in (5). On the other hand, if interest lies in the detection of differentially expressed regions between different time points, inference is performed on each row of a $\frac{q(q-1)}{2}\times T$ matrix ***ZXB***, where ***Z*** is a $\frac{q(q-1)}{2}\times N$ contrast matrix indicating the specific time points to be contrasted. Hence, each row of ***ZXB*** corresponds with one of the $\frac{q(q-1)}{2}$ possible pairwise comparisons between two time points and gives rise to an effect function $F\left\{\beta (t)\right\}$ for each desired comparison. In circadian rhythm designs the sine and the cosine effect functions are combined to give the amplitude ***A***(*t*) of the circadian rhythm per probe position, i.e.

(12) $$F\left\{\beta (t)\right\}=A(t)=\sqrt{\beta_{2}^{2}(t)+\beta_{3}^{2}(t)}.$$

Based on the size of ***A***(*t*) circadian effect regions can be detected. In the case of non-orthogonal designs in multiple-factor studies, there are several possibilities for the choice of $F\left\{\beta (t)\right\}$, depending on the aim of the analysis. The idea remains the same, however.

For each genomic location *t*, $F\left\{\beta (t)\right\}$ is compared to a certain threshold value *δ*which can be chosen freely by the biological researcher. A Bayesian FDR procedure [35] is adopted to evaluate statistical significance. This may be written as

(13) $$\mathrm{FD}R_{F}(t)=P\left[F\left\{\beta (t)\right\}<\delta |Y\right].$$

It basically involves the calculation of a probability mass from a univariate normally distributed random variable if $F\left\{\beta (t)\right\}$ contains only one ***β***(*t*), or from a multivariate normally distributed random variable if $F\left\{\beta (t)\right\}$ contains a linear combination of ***β***(*t*)’s [25]. The variance-covariance matrix is readily available if ***X***is orthogonal. For non-orthogonal designs it can be calculated by

$${\left\{{\left(X_{\mathrm{orth}}^{T}X\right)}^{-1}\right\}}^{T}\text{Var}\left[F\left\{\beta (t)\right\}\right]\left\{{\left(X_{\mathrm{orth}}^{T}X\right)}^{-1}\right\}.$$

For the circadian rhythms design however, this approach is not possible because of the non-linear dependence of ***A***(*t*) on the ***β***(*t*)’s. In this case *FD*_*RF*_(*t*) can be approximated by simulation. In each simulation step we sample from the normal sine and cosine effect functions and calculate _***A***sim_(*t*). *FD*_*RF*_(*t*) is now given by the proportion of simulations for which _***A***sim_(*t*)<*δ*. Specifically for differential expression, (13) is used to detect overexpression at probe *t*, while for detecting underexpression at probe *t* we use

(14) $$\mathrm{FD}R_{F}(t)=P\left[F\left\{\beta (t)\right\}>-\delta |Y\right].$$

## Results and discussion

The use and flexibility of the waveTiling package is illustrated in three case studies for transcriptome analysis with different experimental set-ups.

### Case study 1: Time-course experiment

The first data set consists of a tiling array expression study for identifying the molecular events associated with early leaf development of the plant species *Arabisopsis thaliana*[11]. Unraveling the underlying mechanisms of on one hand the transition from cell division to cell expansion and on the other hand the transition from non-photosynthetic to photosynthetic leaves, was the focus of this study. Trancriptome analysis for six developmental time points (day 8 to day 13) was conducted with AGRONOMICS1 tiling arrays [36], with three biological replicates per time point. Primarily, the researchers were focusing on the detection of differentially expressed regions between any two pairs of developmental time points. This specific study design, however, also allows for the detection of expression regions that change linearly over time. The functions and code used for this case study are described in more detail in the package vignette (see Additional file 1).

#### Pairwise comparison

**Figure 1:**
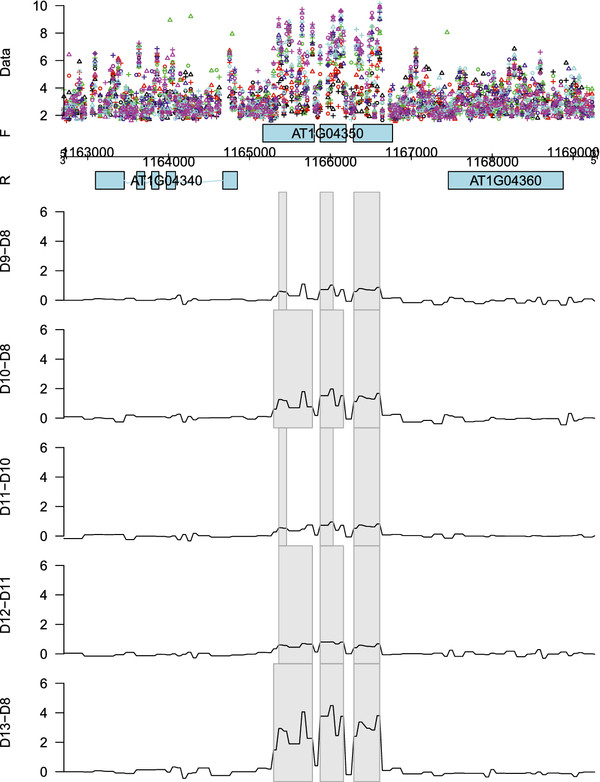
**Pairwise day-to-day differentially expressed genomic region.** Fitted differential expression effect for the genomic region of gene *AT1G04350* on the forward strand of chromosome 1 between selected pairs of developmental time points varying from day 8 (D8) to day 13 (D13). The grey rectangles indicate the detected regions showing a significant differential expression effect. The different replicates are indicated by ∘, + and ▵, while the different days are represented by different colors: black (D8), red (D9), green (D10), blue (D11), cyan (D12) and magenta (D13).

Figure 1 gives an example of a genomic region on chromosome 1 of *Arabidopsis thaliana* found to be differentially expressed between different time points. The threshold value used here was |_log2_(1.2)|. For the most significant time point pairs the detected regions clearly resemble the exons of gene *AT1G04350*, encoding a putative 2-oxoglutarate-dependent dioxygenase (Figure 1).

We evaluate the regions detected by the wavelet-based analysis against the genes produced by the well-established and often used RMA method [37]. This is done by comparing the results of a gene set enrichment analysis based on both methods. By mapping the genomic regions found by the wavelet-based method to the *Arabidopsis thaliana* TAIR9 annotation [38], a list of genes is created for this method. Only genes that showed an overlap of at least 15% with the detected regions were retained. The enrichment analysis as performed with Plaza [39] revealed a strong overlap in the processes detected by both methods. A total of 483 enrichments were identified using both genesets of which 360 common enrichments were shared. The RMA gene list had 75 specific enrichments, while the wavelet-based gene list had 48.

The enrichment analyses revealed a high similarity of genes in common by the two methods for identifying differentially expressed regions of the genome that have previously been annotated. However, we could also discover non-annotated regions that were differentially expressed. We identified a total of 109 unannotated and differentially expressed regions in the genome with a length of at least 200 bp. Selected regions were validated with qRT-PCR to confirm these findings. These regions were chosen based on the following criteria: 

1. Region was not in or near an exon or promoter from an annotated gene.

2. Longer regions containing more differentially expressed probes were preferentially selected.

3. Regions showing homogeneous probe directionality (all probes going in the same direction) across the entire region of differential expression were preferentially selected.

Using these criteria 12 regions were selected and qRT-PCR analysis was performed (see Additional file 2: Table S1). Of the 12 regions, 11 could be confirmed to contain differentially expressed transcripts during the time-course analysis. Only 1 region had no detectable transcriptional products. Log fold changes were calculated for confirming the expression and differential expression, as well as the directionality of the differential expression. From this analysis 9 of the 11 regions showed the same log fold change directionality as previously identified from the tiling arrays, and 2 regions showed opposite log fold change directionality. However, these 2 regions had the lowest log fold changes in the wavelet-based analysis. More details about the methods of enrichment and qRT-PCR analysis can be found in Additional file 2.

#### Linear and quadratic time effects

**Figure 2:**
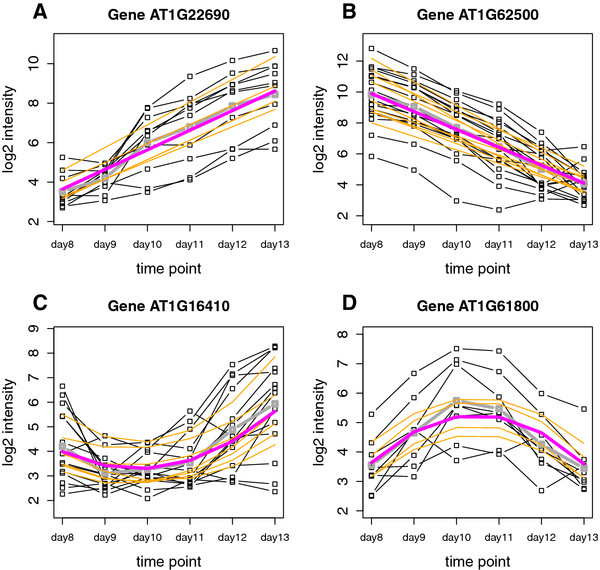
**Gene-wise linear and quadratic effects of transcription levels.** Example plot for two genes showing a linearly increasing (**A**) and decreasing (**B**) mean log_2_ intensity level as a function of the 6 days in the time-course. These genes map to two of the top detected regions with a linear time effect for the forward strand of chromosome 1. The mean of the linear time effect parameter estimates corresponding with the probes in these regions are 1.08 and -1.16 respectively. Plots **C** and **D** give two examples of genes with a strong quadratic effect on the forward strand of chromosome 1. The dotted black lines represent the mean observed log_2_ expression for the probes over the three biological replicates at the different time points. The dotted grey line is the mean observed log_2_ expression over all the probes in the region. The orange lines are the probe-wise fitted log_2_ expression values when only considering the intercept and the linear time effect in the model for the two upper-part genes, and considering the intercept, the linear and the quadratic time effect in the model for the two lower-part genes. The purple line gives the corresponding mean fitted log_2_ expression values at the different time points over all the probes in the region.

In addition to a pairwise comparison analysis, the wavelet-based functional model using the orthogonal polynomial design matrix is also useful for detecting genes with linear and quadratic expression patterns over time. In fact, the estimated parameters now give direct interpretations in terms of the different order time effects. Figure 2 gives some example plots of genes from the forward strand of chromosome 1 with a clear linear or quadratic time effect. From the plots, it is clear that the fitted probe-wise log_2_ intensities at the different time points (orange lines) are squeezed to some extent towards the mean fitted log_2_ intensities over all probes in the whole detected region at these time points (purple line). The main reason for this is that in the wavelet domain strength is borrowed from the neighboring probes in the genomic region to provide a more reliable estimate for each probe-wise effect.

**Figure 3:**
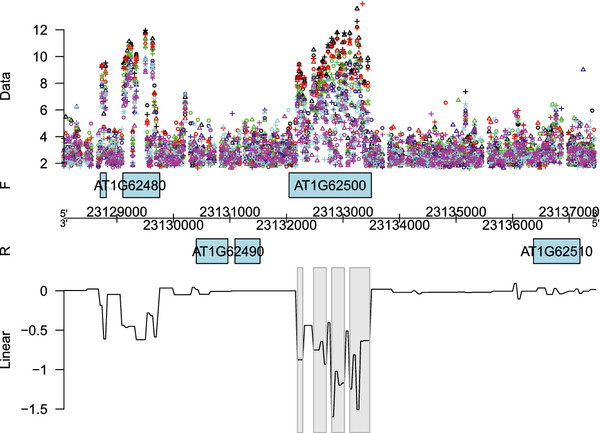
**Genomic region with a linear time effect.** Fitted linear time effect for the genomic region of gene *AT1G62500* on the forward strand of chromosome 1. The different replicates are indicated by ∘, + and ▵, while the different days are represented by different colors: black (D8), red (D9), green (D10), blue (D11), cyan (D12) and magenta (D13). The grey rectangles indicate the detected regions showing a significant linear time effect, while the black line corresponds with the coefficient function of the linear effect. The negative sign of the coefficients implies a decreasing effect over time. More specifically, the effect at probe *t* is ${\hat{\beta }}_{1}(t)\times \mathrm{time}$ .

**Figure 4:**
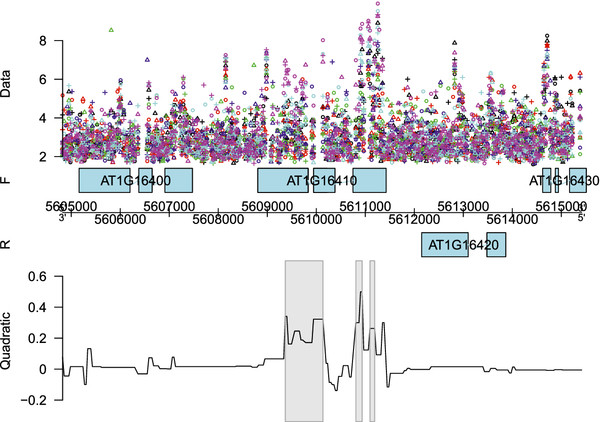
**Genomic region with a quadratic time effect.** Fitted quadratic time effect for the genomic region of gene *AT1G16410* on the forward strand of chromosome 1. The different replicates are indicated by ∘, + and ▵, while the different days are represented by different colors: black (D8), red (D9), green (D10), blue (D11), cyan (D12) and magenta (D13). The grey rectangles indicate the detected regions showing a significant quadratic time effect.

**Figure 5:**
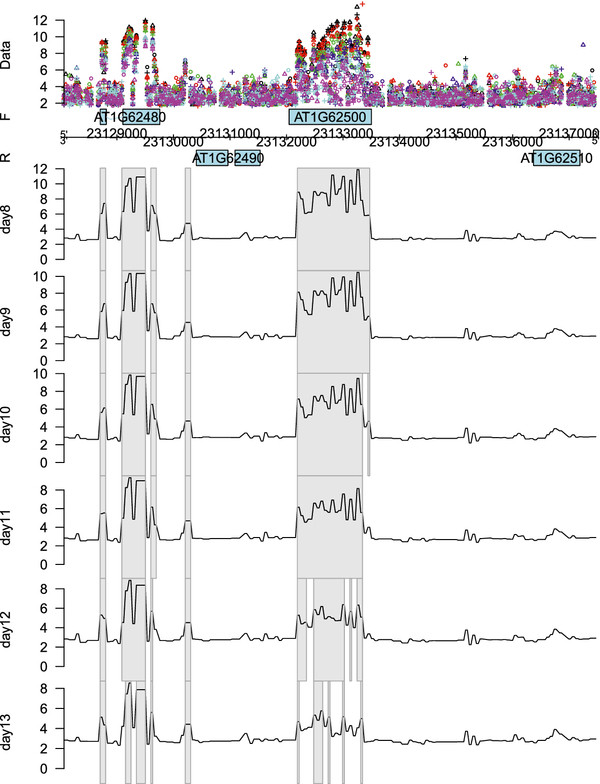
**Day-by-day expression levels for a genomic region show the linear effect.** Fitted log_2_ intensities per time point of the genomic region of gene *AT1G62500* on the forward strand of chromosome 1. The different replicates are indicated by ∘, + and ▵, while the different days are represented by different colors: black (D8), red (D9), green (D10), blue (D11), cyan (D12) and magenta (D13). The grey rectangles indicate the detected regions showing a significant mean expression. The decreasing trend of the fitted log_2_ intensities over the different time points exemplified in Figures 2 and 3 is clearly apparent in this figure.

For two of the genes shown in Figure 2 a more detailed visualization is given of the fitted linear or quadratic time effect along the genomic coordinate of chromosome 1. Figure 3 shows the regions with significant decreasing linear time effects overlapping with gene *AT1G62500*, encoding a putative lipid transfer protein, while Figure 4 shows those regions with a significant quadratic time effect overlapping with gene *AT1G16410*, encoding a cytochrome P450. It is also possible to look at the fitted log_2_ intensities at the different time points. This means that we are still able to perform transcript discovery at each time point separately. Figure 5 gives the corresponding plots for the linearly decreasing gene *AT1G62500*. The trend apparent in the example plots of Figure 2 is also clear from this figure. The grey rectangles in Figure 5 indicate the discovered regions with mean log_2_ intensities significantly above a certain threshold chosen according to the procedure described in [25]. This illustrates that for the discussed models, it is possible to simultaneously detect differentially affected regions between groups as well as transcriptionally active regions for each group - in this case for each day - separately.

### Case study 2: Circadian rhythms

The second case study concerns an expression analysis to examine circadian rhythms in *Arabisopsis thaliana*[13]. It is known that photosynthetic organisms anticipate changes in the daily environment with an internal oscillator, called the circadian clock. The aim of the study was to explore the genome-wide extent of the rhythmic expression patterns governed by this oscillator. In this experiment, 12 samples were collected from *Arabidopsis thaliana* seedlings that were placed under a 12 h light / 12 h dark cycles regime. Every 4 hours 2 samples were taken and hybridized to the Affymetrix AtTile 1.0F and 1.0R tiling arrays. More information about the experiment can be found in [13].

**Figure 6:**
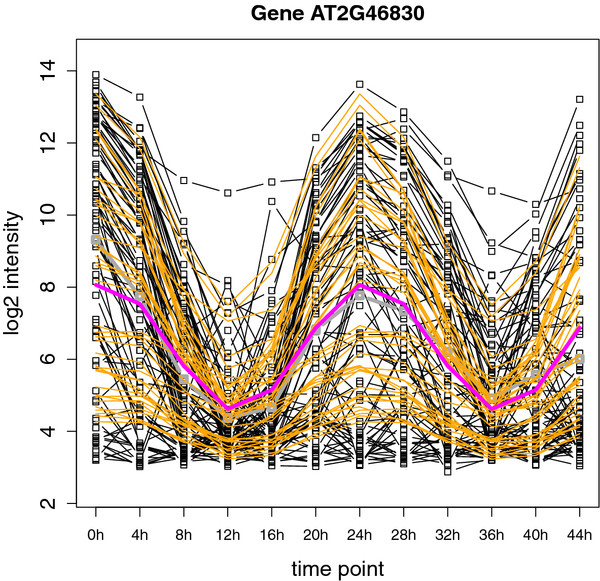
**Gene-wise circadian effect of transcription levels.** Example plot for gene *AT2G46830*, better known as *CIRCADIAN CLOCK ASSOCIATED1*, showing a clear circadian rhythm effect of the mean log_2_ intensity level over the 48h time course. The dotted black lines represent the observed log_2_ expression for the probes at the different time points. The dotted grey line is the mean observed log_2_ expression over all the probes in the region. The orange lines are the probe-wise fitted log_2_ expression values, while the purple line gives the corresponding mean fitted log_2_ expression values at the different time points over all the probes in the region.

**Figure 7:**
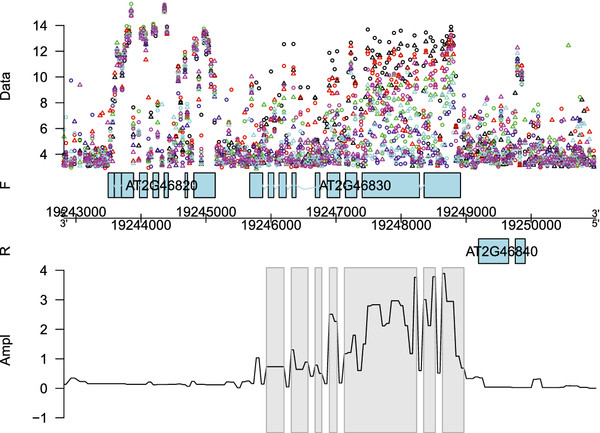
**Genomic region with a circadian effect.** Fitted circadian effect for the genomic region of gene *AT2G46830* on the forward strand of chromosome 2. On the *Y *-axis the amplitude of the circadian rhythm $A(t)=\sqrt{\beta_{2}^{2}(t)+\beta_{3}^{2}(t)}$ is given. The grey rectangles indicate the detected regions showing a significant circadian effect. The different replicates are indicated by ∘ and ▵, while the different samples in the 12 h light / 12 h dark cycles regime are represented by different colorsl.

Figure 6 shows an example of the model fit for gene *AT2G46830* with a clear strong circadian effect. This gene has been previously described and is known under the name *CIRCADIAN CLOCK ASSOCIATED1 (CCA1)*. Besides the circadian effects, no other time-dependent effects are considered in the model. Therefore, the fitted log_2_ intensities for time points at identical moments in the 24h day/night cycle always coincide. This strong circadian effect is confirmed by Figure 7, which shows the fitted effect for the genomic region of *CCA1*. This effect corresponds with the amplitude of the circadian rhythm, $A(t)=\sqrt{\beta_{2}^{2}(t)+\beta_{3}^{2}(t)}$, as estimated by the model.

The performance of the wavelet-based method for circadian rhythms is further tested by examining some specific circadian clock associated genes on the forward strand of the *Arabidopsis thaliana* genome [40]. The genes that we consider here were also reported in [13]. The results are shown in Table 1. All genes show a considerable overlap with the genomic regions for which a circadian effect was detected significantly above the threshold value log_2_(1.1), except *TIME FOR COFFEE* (*AT3G22380*). They also have a quite high maximum estimated effect or amplitude size, except *TIME FOR COFFEE* and *ZEITLUPE* (*AT5G57360*). These latter two genes are the only genes from the list that do not fall within the top 20 genes with the strongest estimated circadian effect for their corresponding chromosome. The gene *TIME FOR COFFEE* is known as a clock gene that does not cycle at the transcriptional level [41]. Hence, it is as expected that both the overlap between detected region and gene annotation, and the effect size are very small. The gene *ZEITLUPE* is reported as having weak rhythms at the transcriptional level [40]. This is confirmed by the low maximum effect size, while still showing a considerable overlap of the significant region with the existing annotation. The results of Table 1 are thus completely in line with what was expected from literature.

**Table 1:** Circadian effect for 9 genes put forward in the Hazen study

Gene ID	Name	Overlap	Max. Eff.	Top 20
*AT1G22770*	*GIGANTEA*	0.529	2.28	yes
*AT1G68050*	*FLAVIN-BINDING KELCH DFB PROTEIN1*	0.867	2.90	yes
*AT2G25930*	*EARLY FLOWERING3*	0.562	1.46	yes
*AT2G46790*	*PSEUDO RESPONSE REGULATOR9*	0.473	1.38	yes
*AT2G46830*	*CIRCADIAN CLOCK ASSOCIATED1*	0.867	3.89	yes
*AT3G22380*	*TIME FOR COFFEE*	0.040	0.06	no
*AT3G46640*	*LUX ARRHYTHMO*	0.717	1.69	yes
*AT5G57360*	*ZEITLUPE*	0.350	0.41	no
*AT5G61380*	*TIMING OF CAB2 EXPRESSION1*	0.797	1.74	yes

Analysis results for 8 circadian clock associated genes and for *TIME FOR COFFEE*, a clock gene that does not cycle at the transcriptional level. These are the genes on the forward strand that were also tested in [13]. *Overlap* indicates the proportion of overlap between the regions detected by the wavelet-based method and the gene annotation; *Max. Eff.* gives the maximum estimated effect or amplitude size for this gene; *Top 20* indicates whether the gene is within the top 20 genes with the strongest circadian effect for the associated chromosome, as produced by the wavelet-based method.

### Case study 3: Non-orthogonal two-factor design

The third data set is used to illustrate the analysis of a two-factor design tiling array experiment. The data are taken from a study of the genome-wide analysis of endogenous abscisic acid (ABA)-mediated transcription in dry and imbibed seeds of *Arabidopsis thaliana*[12]. ABA is a phytohormone that is important for the induction and maintenance of seed dormancy. To understand how endogenous ABA regulates the transcriptome in seeds, whole-genome expression analyses were conducted in two ABA metabolism mutants, an ABA-deficient mutant (*aba2*) and an ABA over-accumulation mutant (*cyp707a1a2a3* triple mutant), and compared to a wild type. This is the first factor in the design. Since endogenous levels of ABA often change drastically during seed imbibition [12], these experiments were done both for dry and for 24-h imbibed seeds. This is the second factor in the design. For each design point, three biological replicates were hybridized using the Affymetrix AtTile 1.0F and 1.0R tiling arrays, resulting in 18 samples. For this case, model (2) can be written as

$$\begin{array}{c} Y_{i}(t)=\beta_{0}(t)+\beta_{1}(t)\;\mathrm{imbibed}+\beta_{2}(t)\;\mathrm{mutant}1\\ \;+\beta_{3}(t)\;\mathrm{mutant}2+\beta_{4}(t)\;\mathrm{imbibed}*\mathrm{mutant}1\\ \;+\beta_{5}(t)\;\mathrm{imbibed}*\mathrm{mutant}2+E_{i}(t), \end{array}$$

where *imbibed*=1 if the seed was imbibed and *imbibed*=0 if the seed was dry, *mutant*1=1 for the *aba2*-mutant and *mutant*1=0 otherwise, and *mutant*2=1 for the *cyp707a1a2a3* triple mutant and *mutant*2=0 otherwise. This model specification implies that the design matrix ***X*** used for this model is

$$X=\left[\begin{array}{cccccc} 1 & 0\; & 0 & 0\; & 0 & 0\\ 1 & 0\; & 1 & 0\; & 0 & 0\\ 1 & 0\; & 0 & 1\; & 0 & 0\\ 1 & 1\; & 0 & 0\; & 0 & 0\\ 1 & 1\; & 1 & 0\; & 1 & 0\\ 1 & 1\; & 0 & 1\; & 0 & 1 \end{array}\right].$$

**Figure 8:**
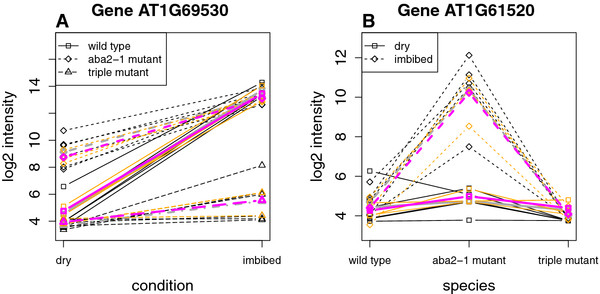
**Gene-wise main and interaction effects of transcription levels for a two-factor model.** Interaction plots for genes *AT1G69530* (**A**) and *AT1G61520* (**B**). The black lines represent the observed log_2_ expression for the probes at the different combinations of the two factor levels. The dotted grey line is corresponding the mean observed log_2_ expression over all the probes in the region. The orange lines are the probe-wise fitted log_2_ expression values, while the purple line gives the corresponding mean fitted log_2_ expression values for all the probes in the region.

Column 1 of ***X***corresponds with an overall mean expression level over all samples. The main imbibition effect is coded in column 2. Note that this corresponds with the imbibition effect for wild types, which is the reference species. Columns 3 and 4 are associated with the main ABA mutation effects, whereas columns 5 and 6 allow to examine an interaction effect between imbibition and ABA mutation statuses. Figure 8 shows two examples plots for representing the model fit for the genes *AT1G69530*, encoding an expansin, and *AT1G61520*, encoding a chlorophyll a/b binding protein, on the forward strand of chromosome 1. Table 2 gives the associated gene-wise mean parameter estimates for these genes. The left panel plot of Figure 8 suggests a larger mean expression level of gene *AT1G69530* for imbibed seeds compared to dry seeds. The increase in mean expression level, however, is larger for wild types than for ABA-related mutants. The increase in mean expression level between imbibed seeds compared to dry seeds is given by ${\hat{\beta }}_{1,\mathrm{gene}}=8.70$ for wild types, while for *aba2* mutants this increase is given by ${\hat{\beta }}_{1,\mathrm{gene}}+{\hat{\beta }}_{4,\mathrm{gene}}=4.36$ and for *cyp707a1a2a3* triple mutants by ${\hat{\beta }}_{1,\mathrm{gene}}+{\hat{\beta }}_{5,\mathrm{gene}}=1.61$. On the right panel of Figure 8 we see an increased mean expression level of gene *AT1G61520* for *aba2* mutants as compared to wild types and *cyp707a1a2a3* triple mutants. In addition, this increase is much stronger for imbibed seeds.

**Table 2:** Two-factor model gene-wise effects

	${\hat{\beta }}_{0,\mathrm{gene}}$	${\hat{\beta }}_{1,\mathrm{gene}}$	${\hat{\beta }}_{2,\mathrm{gene}}$	${\hat{\beta }}_{3,\mathrm{gene}}$	${\hat{\beta }}_{4,\mathrm{gene}}$	${\hat{\beta }}_{5,\mathrm{gene}}$
*AT1G69530*	4.76	8.70	3.98	−0.82	−4.34	−7.09
*AT1G61520*	4.27	0.13	0.72	0.13	5.11	−0.44

Gene-wise mean parameter estimates for genes *AT1G69530* and *AT1G61520*. The estimates indicate a clear interaction effect between *condition* and *species* for these genes, which is further visualized in Figure 8.

## Conclusions

In this paper, we have described the R package waveTiling for model-based analysis of tiling array expression studies with flexible designs. It implements the recently proposed wavelet-based model for transcriptome analysis [25] and extends its applicability towards more complex experimental set-ups. Unlike most currently applied methods, transcriptional activity is modeled at probe-level instead of gene- or exon-level. This probe-wise analysis allows for the detection of transcriptional units in both exonic, intronic and intergenic regions, without prior consultation of existing annotation. By appropriate adaptations of the basic model design matrix it becomes possible to easily analyze the transcriptome for single-factor experiments with more than two biological conditions, to detect linear and quadratic time effects or a circadian rhythm effect in time-course experiments, and to even conduct two- or multiple-factor studies. The package’s use and flexibility are illustrated with three case studies on the reference plant *Arabidopsis thaliana*. These cases show the potential of the package and method to cope with a multitude of study designs and associated specific research questions and still provide reliable results. The waveTiling package will be freely available as part of the Bioconductor project.

### Availability and requirements

• **Project name:** waveTiling

• **Project home page:** http://r-forge.r-project.org/projects/wavetiling/

• **Operating system(s):** Platform independent

• **Programming language:** R

• **Other requirements:** R >= 2.14

• **License:** GNU GPL

• **Any restrictions to use by non-academics:** None

## Competing interests

The authors declare that they have no competing interests.

## Author’s contributions

KDB and LC conceived the study and developed the model. KDB implemented the model, conducted the case studies and statistical analyses and wrote the manuscript. PP helped in the design and implementation of the package. MA conducted the biological validation experiments and analyses and helped write the manuscript. OT and CC took part in several discussions related to the model. DI took part in several discussions regarding the biological data. All authors read and approved the final manuscript.

## Supplementary Material

Additional file 1 **waveTiling package vignette.**Package vignette containing detailed information on how to perform a transcriptome analysis using a wavelet-based functional model with the waveTiling package. The data set of case study 1 (leaf development data) is used in the vignette. Click here for file

Additional file 2 **Methods for biological validation.**Detailed information about the gene set enrichment and qRT-PCR analysis for case study 1 (leaf development data). Click here for file
