# Supplementary material for: Analysis of tiling array expression studies with flexible designs in Bioconductor (waveTiling)
Source: BMC Bioinformatics. 2012 Sep 14;13:234. doi: 10.1186/1471-2105-13-234 (PMC3558343; doi:10.1186/1471-2105-13-234)
Supplement: Additional file 1 — waveTiling package vignette.Package vignette containing detailed information on how to perform a transcriptome analysis using a wavelet-based functional model with the waveTiling package. The data set of case study 1 (leaf development data) is used in the vignette. [file 1471-2105-13-234-S1.pdf]

The *waveTiling* package

Kristof De Beuf

May 15, 2012

Contents

1 Introduction **1**

2 Read in and prepare data for analysis **1**

3 Wavelet-based transcriptome analysis **2**
3.1 Standard analysis flow 2
3.2 Plot function 6
3.3 Accessor functions 9

1 Introduction

In this *waveTiling* package vignette the package's main functionalities to conduct a tiling array trancriptome analysis are illustrated. The package contains an implementation of the basic wavelet-based functional model introduced in [1], and its extensions towards more complex designs described in [3]. The leaf development data set [2] contains genome-wide expression data measured for six developmental time points (day 8 to day 13) on the plant species *Arabidopsis thaliana*. The experiment was conducted with AGRONOMICS1 tiling arrays [4] and contains three biological replicates per time point.

2 Read in and prepare data for analysis

First we have to load the *waveTiling* package and the *waveTilingData* package. The latter contains an *TilingFeatureSet* (leafdev) from the *oligoClasses* package [5] with the expression values for the leaf development experiment, and the TAIR 9 *Arabidopsis thaliana* gene identifier data tair9gff. Make sure to also load the *pd.atdschip.tiling* package which contains the tiling array info to map the probe locations on the array to the exact genomic positions. The *pd.atdschip.tiling* package was created by using the *pdInfoBuilder* package [6], which should also be used to build similar packages for other array designs.

```
> library(waveTiling)
> library(waveTilingData)
> library(pd.atdschip.tiling)
> data(leafdev)
> data(tair9gff)
```

We first change the class to *WaveTilingFeatureSet*, which is used as input for the wavelet-based transcriptome analysis, and add the phenotypic data for this experiment.

```
> leafdev <- as(leafdev,"WaveTilingFeatureSet")
> leafdev <- addPheno(leafdev,noGroups=6,
+         groupNames=c("day8","day9","day10","day11","day12","day13"),
+         replics=rep(3,6))
> leafdev
```

```
WaveTilingFeatureSet (storageMode: lockedEnvironment)
assayData: 6553600 features, 18 samples
  element names: exprs
protocolData
  rowNames: caquinof_20091023_S100_v4.CEL
    caquinof_20091023_S101_v4.CEL ...
    caquinof_20091023_S117_v4.CEL (18 total)
  varLabels: exprs dates
  varMetadata: labelDescription channel
phenoData
  rowNames: day8.1 day8.2 ... day13.3 (18 total)
  varLabels: group replicate
  varMetadata: labelDescription
featureData: none
experimentData: use 'experimentData(object)'
Annotation: pd.atdschip.tiling
```

Before starting the transcriptome analysis, the probes that map to several genomic locations (either PM or MM, or forward and reverse strand) are filtered using `filterOverlap`. This function can also be used if the probes have to be remapped to another version of the genome sequence as the version used for the array design. For instance, the probes on the AGRONOMICS1 array are build based on the TAIR 8 genome, and remapped onto the TAIR 9 sequence. The function needs an argument `BSgenomeObject` available from loading the appropriate *BSgenome* package [7]. The output is an object of class *mapFilterProbe*. After filtering and/or remapping, the expression data are background-corrected and quantile-normalized (`bgCorrQn`). The *mapFilterProbe* `leafdevMapAndFilterTAIR9` is used to make sure only the filtered probes are used in the background correction and normalization step.

```
> library(BSgenome.Athaliana.TAIR.TAIR9)
> # leafdevMapAndFilterTAIR9 <- filterOverlap(leafdev,remap=TRUE,
> #        BSgenomeObject=Athaliana,chrId=1:7,
> #        strand="both",MM=FALSE)
> data(leafdevMapAndFilterTAIR9)
> leafdevBQ <- bgCorrQn(leafdev,useMapFilter=leafdevMapAndFilterTAIR9)
```

3 Wavelet-based transcriptome analysis

3.1 Standard analysis flow

The analysis has to be conducted in a chromosome- and strand-wise manner. First, the wavelet-based model is fitted to the expression data, leading to a *WfmFit*-class object leafdevFit.

```
> chromosome <- 1
> strand <- "forward"
> leafdevFit <- wfm.fit(leafdevBQ,filter.overlap=leafdevMapAndFilterTAIR9,
+         design="time",n.levels=10,
+         chromosome=chromosome,strand=strand,
```

```
+         var.eps="marg",prior="improper",skiplevels=1,
+         save.obs="plot",trace=TRUE)
> leafdevFit

Fitted object from wavelet based functional model - Time Design
Wavelet filter used: haar
Number of wavelet decomposition levels: 10
Number of probes used for estimation: 753664

Genome Info :
        Chromosome: 1
        Strand: forward
        Minimum probe position: 50
        Maximum probe position: 30397589
```

If the redundant probes have been filtered using `filterOverlap` the resulting *mapFilterProbe* class object should be given as an argument `filter.overlap`, to ensure that the expression values are properly linked to the genomic information such as chromosome and strand. In this analysis we use a time-course design (`design`). The number of levels in the wavelet decomposition is 10 (`n.levels`). We use marginal maximum likelihood to estimate the residual variances (`var.eps`) and put an improper prior (`prior`) on the effect functions (see [1]).

Next, the *WfmFit*-class object `leafdevFit` is used as input for the inference function `wfm.inference`. This function outputs the *WfmInf*-class object `leafdevInf` from which transcriptionally active regions of interest, given a chosen threshold value, can be extracted.

```
> delta <- log(1.2,2)
> leafdevInfCompare <- wfm.inference(leafdevFit,
+         contrasts="compare",delta=c("median",delta))
> leafdevInfCompare

object of class 'WfmInf'
```

The `contrasts` argument is used to indicate the type of inference analysis one wants to conduct, e.g. `compare` to detect differentially expressed regions between the different time points. By default, transcriptionally active regions based on the mean expression over all arrays are also given in the output. With the `delta` the threshold value to use in the statistical tests can be set. It is a *vector* with as first element the threshold for the overall mean trancript discovery. This is taken to be the median of the expression values over all arrays in this case. The second element is the threshold for the differential expression analysis. This threshold is equal for each pairwise comparison if the length of `delta` is 2. If one wants to use different thresholds the length of `delta` must be $r+1$ with r the number of pairwise comparisons, where each element is associated with an individual threshold value.

Much information is stored in the *WfmFit*-class and *WfmInf*-class objects. Primarily, we are interested in the genomic regions that are significantly transcriptionally affected according to the research question of interest.

```
> sigGenomeRegionsCompare <- getGenomicRegions(leafdevInfCompare)
> sigGenomeRegionsCompare[[2]]

IRanges of length 439
        start     end width
[1]    118321  118673   353
[2]    145361  145457    97
[3]    163985  164081    97
```

```
[4]      219441   219537    97
[5]      219985   220081    97
[6]      220177   220561   385
[7]      220657   220817   161
[8]      220913   221009    97
[9]      312497   312977   481
...         ...      ...   ...
[431] 29550740 29550836    97
[432] 29916020 29916116    97
[433] 30005524 30005620    97
[434] 30027828 30027924    97
[435] 30040788 30040884    97
[436] 30216949 30217109   161
[437] 30217333 30217429    97
[438] 30217909 30218005    97
[439] 30219061 30219157    97
```

```
> length(sigGenomeRegionsCompare)
```

```
[1] 16
```

The `getGenomicRegions` accessor outputs a `list` of `IRanges` objects [8] denoting the start and end position of each significant region. The first element in the `list` always gives the significant regions for the mean expression over all arrays (transcript discovery). Elements 2 to 16 in `sigGenomeRegions` give the differentially expressed regions between any pair of contrasts between different time points. The order is always 2-1, 3-1, 3-2, 4-1,... Hence, `sigGenomeRegions[[2]]` gives the differentially expressed regions between time point 2 and time point 1.

If an annotation file containing gene identifiers is available, we can extract both significantly affected genes with `getSigGenes`, and the non-annotated regions with `getNonAnnotatedRegions`. Both functions output a *list* of *GRanges* objects [9].

```
> sigGenesCompare <- getSigGenes(fit=leafdevFit,inf=leafdevInfCompare,annoFile=tair9gff)
> head(sigGenesCompare[[2]])
```

```
GRanges with 6 ranges and 6 elementMetadata cols:
      seqnames           ranges strand |     feature          id
         <Rle>        <IRanges>  <Rle> | <character> <character>
  [1]        1 [116943, 118764]      + |        gene   AT1G01300
  [2]        1 [143564, 145684]      + |        gene   AT1G01370
  [3]        1 [163419, 166239]      + |        gene   AT1G01448
  [4]        1 [218994, 221286]      + |        gene   AT1G01600
  [5]        1 [218994, 221286]      + |        gene   AT1G01600
  [6]        1 [218994, 221286]      + |        gene   AT1G01600
          regNo percOverGene percOverReg totPercOverGene
      <integer>    <numeric>   <numeric>       <numeric>
  [1]         1    19.374314         100       19.374314
  [2]         2     4.573314         100        4.573314
  [3]         3     3.438497         100        3.438497
  [4]         4     4.230266         100       36.502399
  [5]         5     4.230266         100       36.502399
  [6]         6    16.790231         100       36.502399
  ---
```

```
  seqlengths:
    1  2  3  4  5  6  7
   NA NA NA NA NA NA NA
```

```
> nonAnnoCompare <- getNonAnnotatedRegions(fit=leafdevFit,inf=leafdevInfCompare,
+          annoFile=tair9gff)
> head(nonAnnoCompare[[2]])
```

```
GRanges with 6 ranges and 0 elementMetadata cols:
      seqnames       ranges strand
         <Rle>    <IRanges>  <Rle>
  [1]        1 [ 113,  241]      +
  [2]        1 [ 338,  977]      +
  [3]        1 [1585, 1713]      +
  [4]        1 [1810, 2097]      +
  [5]        1 [2289, 2834]      +
  [6]        1 [3121, 3281]      +
  ---
  seqlengths:
    1
   NA
```

Using the same *WfmFit*-object `leafdevFit`, we can run the analysis to analyze transcriptional time effects (`leafDevInfTimeEffect`) and have a look at time-wise trancriptionally active regions (`leafdevInfMeans`).

```
> leafdevInfTimeEffect <- wfm.inference(leafdevFit,contrasts="effects",
+          delta=c("median",2,0.2,0.2,0.2,0.2))
> leafdevInfMeans <- wfm.inference(leafdevFit,contrasts="means",
+          delta=4,minRunPos=30,minRunProbe=-1)
```

Besides the available standard design analyses given by the `design` argument in the `wfm.fit` function and the `contrasts` argument in the `wfm.inference`, it is also possible to provide custom design and contrast matrices in the *waveTiling* package. This `custom` design is illustrated based on the polynomial contrast matrix used in a time-course analysis.

```
> custDes <- matrix(0,nrow=18,ncol=6)
> orderedFactor <- factor(1:6,ordered=TRUE)
> desPoly <- lm(rnorm(6)~orderedFactor,x=TRUE)$x
> custDes[,1] <- 1
> custDes[,2:6] <- apply(desPoly[,2:6],2,rep,getReplics(leafdevBQ))
> custDes
```

```
      [,1]       [,2]       [,3]       [,4]       [,5]        [,6]
 [1,]    1 -0.5976143  0.5455447 -0.3726780  0.1889822 -0.06299408
 [2,]    1 -0.5976143  0.5455447 -0.3726780  0.1889822 -0.06299408
 [3,]    1 -0.5976143  0.5455447 -0.3726780  0.1889822 -0.06299408
 [4,]    1 -0.3585686 -0.1091089  0.5217492 -0.5669467  0.31497039
 [5,]    1 -0.3585686 -0.1091089  0.5217492 -0.5669467  0.31497039
 [6,]    1 -0.3585686 -0.1091089  0.5217492 -0.5669467  0.31497039
 [7,]    1 -0.1195229 -0.4364358  0.2981424  0.3779645 -0.62994079
 [8,]    1 -0.1195229 -0.4364358  0.2981424  0.3779645 -0.62994079
 [9,]    1 -0.1195229 -0.4364358  0.2981424  0.3779645 -0.62994079
[10,]    1  0.1195229 -0.4364358 -0.2981424  0.3779645  0.62994079
```

```
[11,]    1  0.1195229 -0.4364358 -0.2981424  0.3779645  0.62994079
[12,]    1  0.1195229 -0.4364358 -0.2981424  0.3779645  0.62994079
[13,]    1  0.3585686 -0.1091089 -0.5217492 -0.5669467 -0.31497039
[14,]    1  0.3585686 -0.1091089 -0.5217492 -0.5669467 -0.31497039
[15,]    1  0.3585686 -0.1091089 -0.5217492 -0.5669467 -0.31497039
[16,]    1  0.5976143  0.5455447  0.3726780  0.1889822  0.06299408
[17,]    1  0.5976143  0.5455447  0.3726780  0.1889822  0.06299408
[18,]    1  0.5976143  0.5455447  0.3726780  0.1889822  0.06299408
```

```
> leafdevFitCustom <- wfm.fit(leafdevBQ,filter.overlap=leafdevMapAndFilterTAIR9,
+         design="custom",design.matrix=custDes,n.levels=10,
+         chromosome=chromosome,strand=strand,var.eps="marg",
+         prior="improper",skiplevels=1,save.obs="plot",trace=TRUE)
> noGroups <- getNoGroups(leafdevBQ)
> myContrastMat <- matrix(0,nrow=noGroups*(noGroups-1)/2,ncol=noGroups)
> hlp1 <- rep(2:noGroups,1:(noGroups-1))
> hlp2 <- unlist(sapply(1:(noGroups-1),function(x) seq(1:x)))
> for (i in 1:nrow(myContrastMat))
+ {
+         myContrastMat[i,hlp1[i]] <- 1
+         myContrastMat[i,hlp2[i]] <- -1
+ }
> myContrastMat
```

```
      [,1] [,2] [,3] [,4] [,5] [,6]
 [1,]   -1    1    0    0    0    0
 [2,]   -1    0    1    0    0    0
 [3,]    0   -1    1    0    0    0
 [4,]   -1    0    0    1    0    0
 [5,]    0   -1    0    1    0    0
 [6,]    0    0   -1    1    0    0
 [7,]   -1    0    0    0    1    0
 [8,]    0   -1    0    0    1    0
 [9,]    0    0   -1    0    1    0
[10,]    0    0    0   -1    1    0
[11,]   -1    0    0    0    0    1
[12,]    0   -1    0    0    0    1
[13,]    0    0   -1    0    0    1
[14,]    0    0    0   -1    0    1
[15,]    0    0    0    0   -1    1
```

```
> leafdevInfCustom <- wfm.inference(leafdevFitCustom,contrast.matrix=myContrastMat,
+         delta=c("median",log(1.2,2)))
```

3.2 Plot function

Plots can be made very easily using the `plotWfm` function which needs both the *WfmFit*- and *WfmInf*-class objects as input. It also needs an appropriate annotation file. The plot function makes use of the implementations in the *GenomeGraphs*-package [10].

```
> gene1 <- tair9gff[tair9gff$ID %in% "AT1G04350",]
> start <- gene1$start-2500
```

```
> end <- gene1$end+2500
> plotWfm(fit=leafdevFit,inf=leafdevInfCompare,
+         annoFile=gene1,minPos=start,maxPos=end,
+         two.strand=TRUE,plotData=TRUE,
+         plotMean=FALSE,tracks=c(1,2,6,10,11))
```

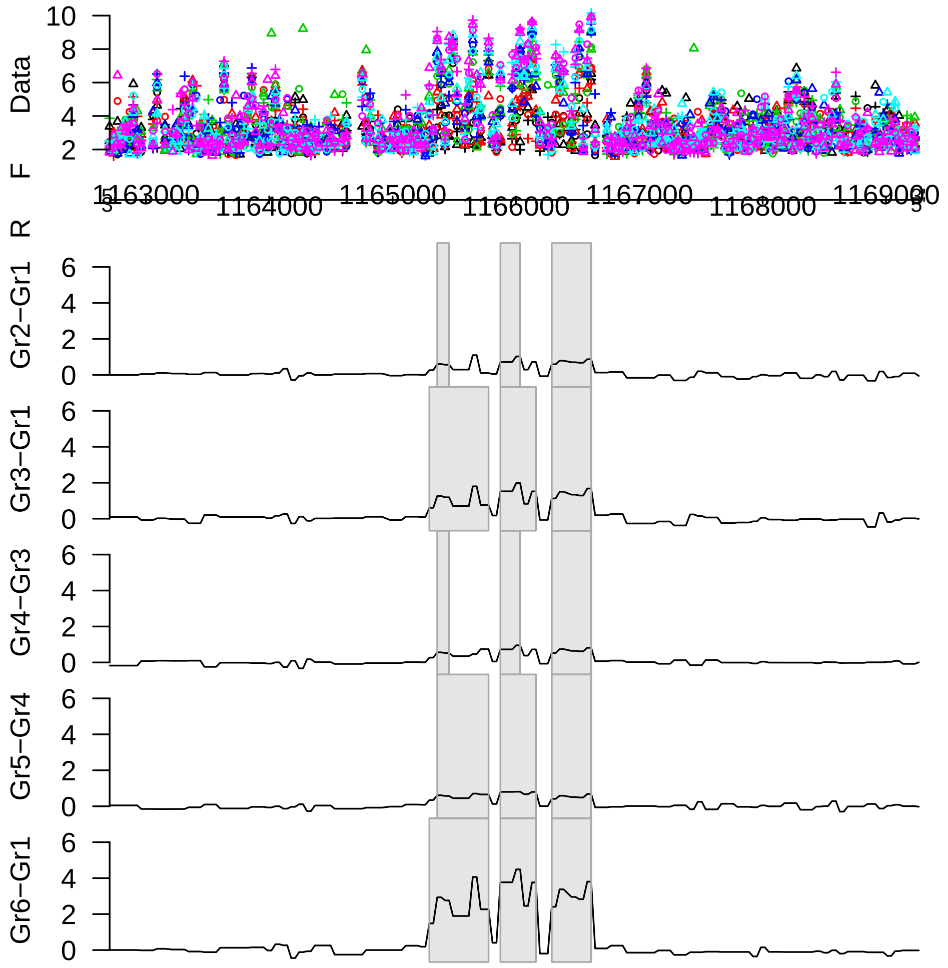


```
> gene2 <- tair9gff[tair9gff$ID %in% "AT1G62500",]
> start <- gene2$start-4000
> end <- gene2$end+4000
> plotWfm(fit=leafdevFit,inf=leafdevInfTimeEffect,
+         annoFile=gene2,minPos=start,maxPos=end,
+         two.strand=TRUE,plotData=TRUE,
+         plotMean=FALSE,tracks=1)
```

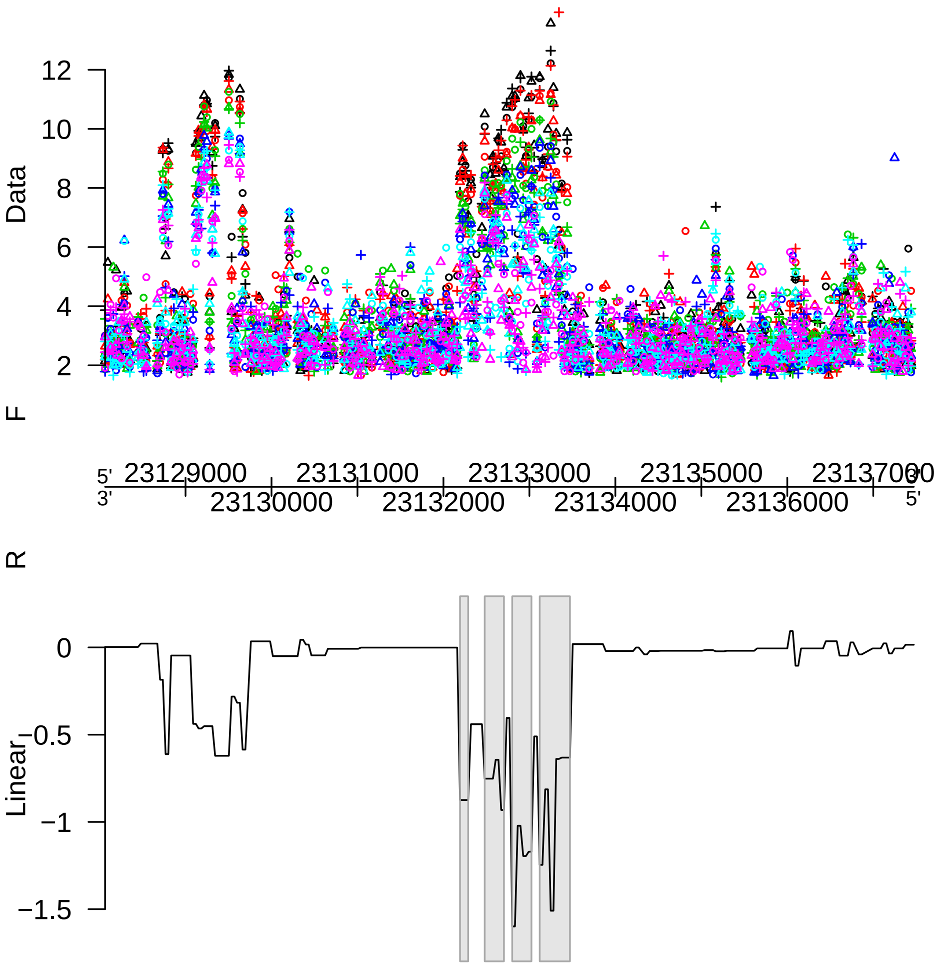


```
> plotWfm(fit=leafdevFit,inf=leafdevInfMeans,
+          annoFile=gene2,minPos=start,maxPos=end,
+          two.strand=TRUE,plotData=TRUE,
+          plotMean=FALSE,tracks=1:6)
```

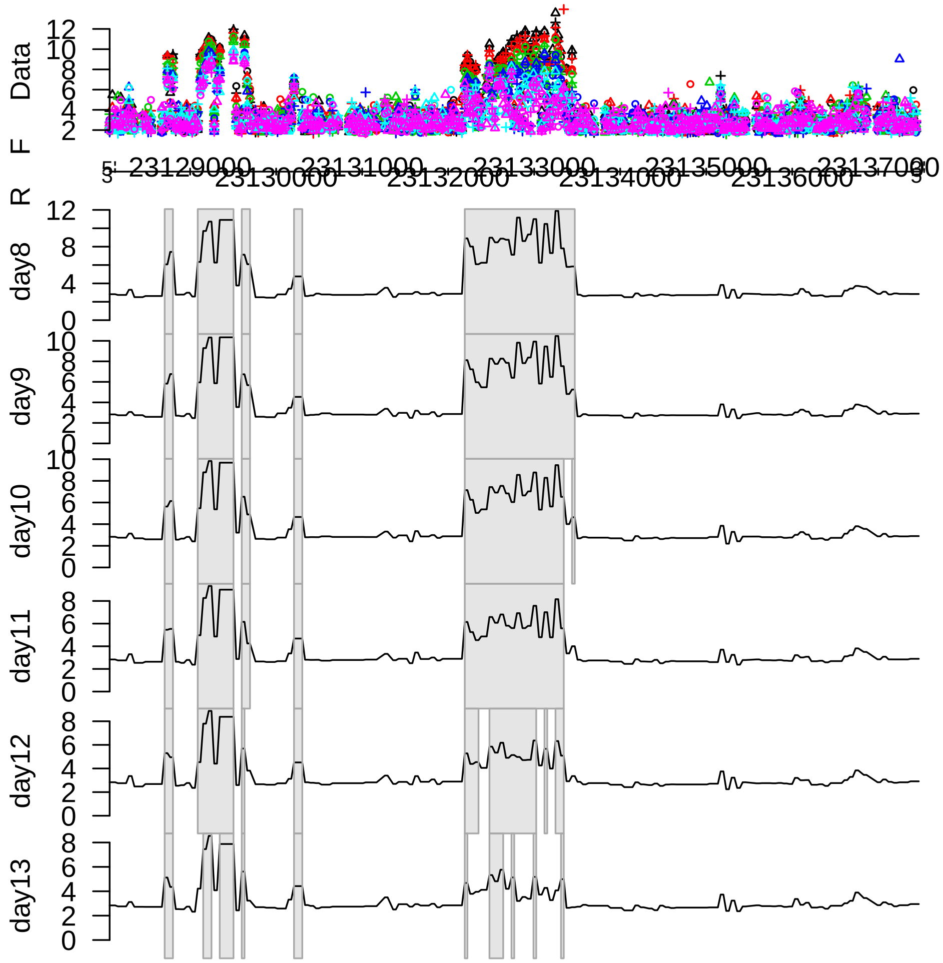

3.3 Accessor functions

There are a number of accessor functions available that are not necessarily needed to run a standard trancriptome analysis, but still can extract useful information from the *WfmFit*- and *WfmInf*-class objects. Some of the more interesting ones are illustrated below. For a complete overview, consult the package's help pages.

```
> getGenomeInfo(leafdevFit)

Genome Info :
        Chromosome: 1
        Strand: forward
        Minimum probe position: 50
        Maximum probe position: 30397589

> dataOrigSpace <- getDataOrigSpace(leafdevFit)
> dim(dataOrigSpace)

[1]     18 753664

> dataOrigSpace[1:8,1:8]
```

```
         307998   698838   619075   234395   361615   533643   672225
day8.1  2.174546 3.052743 2.138756 2.367355 2.138756 5.000700 2.558657
day8.2  1.814251 2.328446 4.165652 3.734485 2.892113 5.173654 3.805115
day8.3  2.803171 2.196780 3.676953 3.380027 3.616066 3.798155 2.536061
day9.1  2.006381 2.176768 3.484187 2.773344 3.184647 5.014818 3.542103
day9.2  2.280221 2.486904 2.860395 3.865426 2.380023 4.238844 2.536061
day9.3  2.834020 2.600848 2.550766 2.954089 3.390393 4.836999 2.655298
day10.1 3.070930 2.857383 3.602468 2.803171 2.702066 6.216891 3.359889
day10.2 2.213182 2.957153 3.825710 3.181483 2.380023 3.667084 2.380023
          735748
day8.1  3.132112
day8.2  2.146153
day8.3  2.695701
day9.1  2.416558
day9.2  4.140161
day9.3  2.550766
day10.1 4.406534
day10.2 2.213182
```

```
> dataWaveletSpace <- getDataWaveletSpace(leafdevFit)
> dim(dataWaveletSpace)
```

```
[1]     18 753664
```

```
> dataWaveletSpace[1:8,1:8]
```

```
              [,1]       [,2]      [,3]        [,4]       [,5]
day8.1   0.6209791  0.1616440 2.0236997  0.40549360  0.6196431
day8.2   0.3635910 -0.3048809 1.6132925 -1.17306372 -0.4977551
day8.3  -0.4287828 -0.2099587 0.1287563  0.11288256 -0.9997125
day9.1   0.1204821 -0.5026421 1.2941265 -0.79588033  0.1552622
day9.2   0.1461465  0.7106642 1.3143853  1.13427053 -0.0701715
day9.3  -0.1648775  0.2851923 1.0229051 -0.07391526 -0.5789659
day10.1 -0.1510001 -0.5651883 2.4853564  0.74008982  0.3803080
day10.2  0.5260667 -0.4555375 0.9100900 -0.11797403 -0.5671088
               [,6]        [,7]       [,8]
day8.1   0.54089690  0.99127802  0.5479831
day8.2  -0.08285642  0.83747549 -0.1653711
day8.3   0.76416907  0.15395435  0.3799351
day9.1   0.16698056  0.67777308 -1.1497952
day9.2   0.03655212  0.53390384 -1.4254594
day9.3  -0.40229994  1.50563770 -0.1675201
day10.1  1.97672325 -0.08831259  0.8829002
day10.2 -0.43654967 -0.19902948  0.1085624
```

```
> getDesignMatrix(leafdevFit)
```

```
      [,1]       [,2]       [,3]       [,4]       [,5]        [,6]
 [1,]    1 -0.5976143  0.5455447 -0.3726780  0.1889822 -0.06299408
 [2,]    1 -0.5976143  0.5455447 -0.3726780  0.1889822 -0.06299408
 [3,]    1 -0.5976143  0.5455447 -0.3726780  0.1889822 -0.06299408
 [4,]    1 -0.3585686 -0.1091089  0.5217492 -0.5669467  0.31497039
```

```
 [5,]    1 -0.3585686 -0.1091089  0.5217492 -0.5669467  0.31497039
 [6,]    1 -0.3585686 -0.1091089  0.5217492 -0.5669467  0.31497039
 [7,]    1 -0.1195229 -0.4364358  0.2981424  0.3779645 -0.62994079
 [8,]    1 -0.1195229 -0.4364358  0.2981424  0.3779645 -0.62994079
 [9,]    1 -0.1195229 -0.4364358  0.2981424  0.3779645 -0.62994079
[10,]    1  0.1195229 -0.4364358 -0.2981424  0.3779645  0.62994079
[11,]    1  0.1195229 -0.4364358 -0.2981424  0.3779645  0.62994079
[12,]    1  0.1195229 -0.4364358 -0.2981424  0.3779645  0.62994079
[13,]    1  0.3585686 -0.1091089 -0.5217492 -0.5669467 -0.31497039
[14,]    1  0.3585686 -0.1091089 -0.5217492 -0.5669467 -0.31497039
[15,]    1  0.3585686 -0.1091089 -0.5217492 -0.5669467 -0.31497039
[16,]    1  0.5976143  0.5455447  0.3726780  0.1889822  0.06299408
[17,]    1  0.5976143  0.5455447  0.3726780  0.1889822  0.06299408
[18,]    1  0.5976143  0.5455447  0.3726780  0.1889822  0.06299408
```

```
> probepos <- getProbePosition(leafdevFit)
> length(probepos)
```

```
[1] 753664
```

```
> head(probepos)
```

```
[1]  50  82 113 177 209 241
```

```
> effects <- getEff(leafdevInfCompare)
> dim(effects)
```

```
[1]     16 753664
```

```
> effects[1:8,1:8]
```

```
             [,1]          [,2]          [,3]          [,4]
[1,]  2.6268519034  2.6268519034  3.0971474646  3.0971474646
[2,] -0.0340036871 -0.0340036871 -0.0340036871 -0.0340036871
[3,]  0.0050326662  0.0050326662  0.0050326662  0.0050326662
[4,]  0.0390363534  0.0390363534  0.0390363534  0.0390363534
[5,] -0.0338712443 -0.0338712443 -0.0338712443 -0.0338712443
[6,]  0.0001324428  0.0001324428  0.0001324428  0.0001324428
[7,] -0.0389039105 -0.0389039105 -0.0389039105 -0.0389039105
[8,] -0.0386280279 -0.0386280279 -0.0386280279 -0.0386280279
             [,5]          [,6]          [,7]          [,8]
[1,]  3.6378117746  3.6378117746  2.7830456751  2.7830456751
[2,] -0.0340036871 -0.0340036871 -0.0340036871 -0.0340036871
[3,]  0.0050326662  0.0050326662  0.0050326662  0.0050326662
[4,]  0.0390363534  0.0390363534  0.0390363534  0.0390363534
[5,] -0.0338712443 -0.0338712443 -0.0338712443 -0.0338712443
[6,]  0.0001324428  0.0001324428  0.0001324428  0.0001324428
[7,] -0.0389039105 -0.0389039105 -0.0389039105 -0.0389039105
[8,] -0.0386280279 -0.0386280279 -0.0386280279 -0.0386280279
```

```
> fdrs <- getFDR(leafdevInfCompare)
> dim(fdrs)
```

```
[1]     16 753664
```

```
> fdrs[1:8,1:8]
          [,1]      [,2]        [,3]        [,4]        [,5]
[1,] 0.9346885 0.9346885 0.001058567 0.001058567 1.05712e-15
[2,] 1.0000000 1.0000000 1.000000000 1.000000000 1.00000e+00
[3,] 1.0000000 1.0000000 1.000000000 1.000000000 1.00000e+00
[4,] 1.0000000 1.0000000 1.000000000 1.000000000 1.00000e+00
[5,] 1.0000000 1.0000000 1.000000000 1.000000000 1.00000e+00
[6,] 1.0000000 1.0000000 1.000000000 1.000000000 1.00000e+00
[7,] 1.0000000 1.0000000 1.000000000 1.000000000 1.00000e+00
[8,] 1.0000000 1.0000000 1.000000000 1.000000000 1.00000e+00
            [,6]      [,7]      [,8]
[1,] 1.05712e-15 0.4957921 0.4957921
[2,] 1.00000e+00 1.0000000 1.0000000
[3,] 1.00000e+00 1.0000000 1.0000000
[4,] 1.00000e+00 1.0000000 1.0000000
[5,] 1.00000e+00 1.0000000 1.0000000
[6,] 1.00000e+00 1.0000000 1.0000000
[7,] 1.00000e+00 1.0000000 1.0000000
[8,] 1.00000e+00 1.0000000 1.0000000
```

References

[1] Clement L, De Beuf K, Thas O, Vuylsteke M, Irizarry RA, and Crainiceanu CM (2012) Fast wavelet based functional models for transcriptome analysis with tiling arrays. *Statistical Applications in Genetics and Molecular Biology*, **11**, Iss. 1, Article 4.

[2] Andriankaja M, Dhondt S, De Bodt S, Vanhaeren H, Coppens F, et al. (2012) Exit from proliferation during leaf development in arabidopsis thaliana: A not-so-gradual process. *Developmental Cell*, **22**, 64–78.

[3] De Beuf K, Pipelers, P, Andriankaja M, Thas O., Inze D, Crainiceanu CM, and Clement L (2012). Model-based Analysis of Tiling Array Expression Studies with Flexible Designs (waveTiling). *Technical document.*

[4] Rehrauer H, Aquino C, Gruissem W, Henz S, Hilson P, Laubinger S, Naouar N, Patrignani A, Rombauts S, Shu H, et al. (2010) AGRONOMICS1: a new resource for Arabidopsis transcriptome profiling. *Plant Physiology*, **152**, 487–499.

[5] Carvalho B and Scharpf R oligoClasses: Classes for high-throughput arrays supported by oligo and crlmm. [R package version 1.18.0]

[6] Falcon S and Carvalho B with contributions by Vince Carey and Matt Settles and Kristof de Beuf pdInfoBuilder: Platform Design Information Package Builder. [R package version 1.20.0]

[7] Pages H BSgenome: Infrastructure for Biostrings-based genome data packages. [R package version 1.24.0]

[8] Pages H, Aboyoun P, and Lawrence M IRanges: Infrastructure for manipulating intervals on sequences. [R package version 1.14.2]

[9] Aboyoun P, Pages H, and Lawrence M GenomicRanges: Representation and manipulation of genomic intervals. [R package version 1.8.3]

[10] Durinck S and Bullard J GenomeGraphs: Plotting genomic information from Ensembl. [R package version 1.16.0]
