# Supplementary material for: Analysis of tiling array expression studies with flexible designs in Bioconductor (waveTiling)
Source: BMC Bioinformatics. 2012 Sep 14;13:234. doi: 10.1186/1471-2105-13-234 (PMC3558343; doi:10.1186/1471-2105-13-234)
Supplement: Additional file 2 — Methods for biological validation.Detailed information about the gene set enrichment and qRT-PCR analysis for case study 1 (leaf development data). [file 1471-2105-13-234-S2.pdf]

Supplementary information

Methods for biological validation

Enrichment analysis

Enrichment analysis was performed by uploading the genesets into PLAZA (Proost et al., 2009) using the PLAZA gene identifiers conversion tool. Gene lists were then subjected to enrichment analysis using the PLAZA GO enrichment tool and a p-value cut-off of 0.05.

qRT-PCR validation of unannotated genomic regions

cDNA synthesis was carried out using 500ng of total RNA and the iScript cDNA Synthesis Kit (BIO-RAD) according to manufacturers instructions. Primers were designed using Primer3 (Rozen and Skaletsky, 2000). The qRT-PCR was performed in 384-well plates using a LightCycler 480 (Roche Diagnostics) and LightCycler 480 SYBR Green-1 Master (Roche) according to manufacturers instructions. All melting curves were checked to confirm primer specificity. Genes were normalized against the average of 3 housekeeping genes (HK) (*AT1G13320*, *AT2G32170*, and *AT2G28390*) (Czechowski et al., 2005). Ct is determined as the number of cycles when SYBR Green fluorescence reaches a specified value during the exponential phase of cDNA amplification. qRT-PCR log fold changes were calculated as follows:

$$\begin{aligned}\text{Log Fold Change} &= (-\Delta\Delta Ct)\\ &= (-1)\left[(Ct_{\text{gene 1, sample 1}} - Ct_{\text{average HK sample 1}})\right.\\ &\quad \left.-(Ct_{\text{gene 1, sample 2}} - Ct_{\text{average HK sample 2}})\right].\end{aligned}$$

The non-annotated regions chosen for validation can be found in Table 1.

chromosome	strand orientation	start	end
5	forward	1042594	1042866
4	reverse	7642124	7643140
1	forward	2407636	2408052
1	forward	17392521	17392937
1	reverse	17391545	17391961
1	reverse	16115545	16115929
5	forward	1042610	1042866
2	forward	17224523	17224779
1	reverse	29649701	29649956
2	reverse	3326499	3326724
4	forward	10180294	10180518
4	forward	13716555	13716779

Table 1: Non-annotated regions chosen for qRT-PCR validation.

References

Czechowski, T., Stitt, M., Altmann, T., Udvardi, M. K., and Scheible, W. R. (2005). Genome-wide identification and testing of superior reference genes for transcript normalization in arabidopsis. *Plant Physiology*, 139(1):5–17.

Proost, S., Van Bel, M., Stercka, L., Billiaua, K., Van Parysa, T., Van de Peer, Y., and Vandepoele, K. (2009). Plaza: A comparative genomics resource to study gene and genome evolution in plants. *The Plant Cell*, 21(12):3718–3731.

Rozen, S. and Skaletsky, H. (2000). Primer3 on the www for general users and for biologist programmers. In Krawetz, S. and Misener, S., editors, *Bioinformatics Methods and Protocols: Methods in Molecular Biology*, pages 365–386. Humana Press, Totowa, NJ.
